# Effect of a Dual Task on Postural Control in Dyslexic Children

**DOI:** 10.1371/journal.pone.0035301

**Published:** 2012-04-16

**Authors:** Agathe Legrand, Emmanuel Bui-Quoc, Karine Doré-Mazars, Christelle Lemoine, Christophe-Loïc Gérard, Maria Pia Bucci

**Affiliations:** 1 Laboratoire de Psychologie et Neuropsychologie Cognitives, IUPDP, Université Paris Descartes, Sorbonne Paris Cité, Institut de Psychologie, Boulogne Billancourt, France; 2 Service d'Ophtalmologie, Hôpital Robert Debré, Paris, France; 3 Institut Universitaire de France, Paris, France; 4 Service de Psychopathologie de l'enfant et de l'adolescent, Hôpital Robert Debré, Paris, France; Ecole Normale Supérieure, France

## Abstract

Several studies have examined postural control in dyslexic children; however, their results were inconclusive. This study investigated the effect of a dual task on postural stability in dyslexic children. Eighteen dyslexic children (mean age 10.3±1.2 years) were compared with eighteen non-dyslexic children of similar age. Postural stability was recorded with a platform (TechnoConcept®) while the child, in separate sessions, made reflex horizontal and vertical saccades of 10° of amplitude, and read a text silently. We measured the surface and the mean speed of the center of pressure (CoP). Reading performance was assessed by counting the number of words read during postural measures. Both groups of children were more stable while performing saccades than while reading a text. Furthermore, dyslexic children were significantly more unstable than non-dyslexic children, especially during the reading task. Finally, the number of words read by dyslexic children was significantly lower than that of non-dyslexic children and, in contrast to the non-dyslexic children. In line with the U-shaped non-linear interaction model, we suggest that the attention consumed by the reading task could be responsible for the loss of postural control in both groups of children. The postural instability observed in dyslexic children supports the hypothesis that such children have a lack of integration of multiple sensorimotor inputs.

## Introduction

Several studies have explored the effect of a dual task on postural control [1]–[3], and it has been found that cognitive tasks do influence postural stability. Lacour et al. [4] proposed three models for explaining how cognitive tasks affect postural control. In the cross-domain competition model, attention is shared between the postural performance and the cognitive task; consequently, postural control in a dual-task paradigm is impaired compared to in a single postural task. While several studies of normal adults support this idea [5]–[7], other investigations have reported opposite findings [8]–[9]. The nature of the cognitive task used is most likely responsible for the different results. In line with this thinking, the U-shaped non-linear interaction model suggests that the cognitive demand of the secondary task can either improve or diminish postural stability. For instance, an easy cognitive task can shift the focus of attention away from postural control, leading to an improved automatic postural performance [10]–[12]. However, Huxhold et al. [13] reported that increasing the cognitive demand induces an increase in postural instability. Finally, the third model, the task prioritization model, hypothesizes that subjects prioritize postural control over cognitive activity. This strategy is often used by older people [14] and in cases of pathologies [15].

It should be noted that studies dealing with children's postural control while they perform a cognitive task are quite recent. Indeed, Blanchard et al. [16] studied the effects of a cognitive task on balance in children (between 8 and 10 years old) and reported an improvement in postural stability, in terms of smaller sway variability, when children are performing a task such as counting backward or reading a sentence compared to that recorded when they look at an image. However, they observed that the center of pressure (CoP) path was longer, suggesting that children and adults use different strategies. In contrast, Schmid et al. [17], using a similar task (mentally counting backwards task executed silently), showed a strong perturbation of postural stability in 9-year-old children.

Postural instability has been found to increase in 7-year-old children when they were asked to perform a modified Stroop task [18]; the same phenomenon was found in 5-year-old children, both normal and with developmental coordination disorders, while they were naming simple objects that appeared consecutively on a screen [19]. In a recent study, Olivier et al. [20] examined age-related differences in interference with postural control by cognitive tasks, and showed that the mature level of attention is reached at about 11 years old. In line with the U-shaped non-linear model [4], these authors suggested that there are two independent attentional mechanisms, one for controlling postural control and the other responsible for the cognitive task; two such mechanisms could interfere with each other depending on the difficulty of the tested condition.

The present study examines the question of whether a dual task can influence postural stability in 10-year-old dyslexic children. Dyslexia is a neurobiological disorder characterized by a difficulty in reading acquisition despite adequate intelligence, conventional education and motivation [21]. Different theories have been suggested for the etiology of dyslexia. The phonological theory make the hypothesis that dyslexia is a direct consequence of cognitive deficits specific to the representation and processing of speech sounds [22]–[24]. Some authors considered that the phonology have a secondary place and that dyslexia is most likely a sensori motor deficit related to: auditory [25], visual [26], cerebellar [27] and the magnocellular impairment [28]. In dyslexic population the oculomotor behavior during reading task has been observed in several studies, and some authors [29]–[31] showed a different oculomotor pattern during reading with respect to non dyslexic population: longer fixation durations, more frequent fixations, shorter saccade amplitudes and more regressive saccades has been found in dyslexic participants.

Nicolson et al. [32], who reported motor coordination and balance deficits in dyslexic children, advanced the hypothesis that dyslexia is characterized by a cerebellar deficiency. Several subsequent studies were done on the issue, producing conflicting results. For instance, several authors [24], [33]–[34] reported impaired postural control in dyslexia but only in some cases, suggesting that the impairment was not strictly correlated with dyslexia but also with other types of developmental disorders [35].

In a dual-task condition, Nicolson and Fawcett [36] reported that postural stability decreased in dyslexic children, suggesting that this population needs to invest more attentional resources than non-dyslexic children to control their balance. Recent studies [37]–[38] have suggested that dyslexic children have a postural deficiency syndrome constituting an alteration of postural equilibrium accompanied by a deficit affecting proprioceptive and visual information. A cognitive task, such as reading single words, impairs postural stability in dyslexic children [37]. Interestingly, a vibration of the ankle muscles impaired stability more strongly in dyslexic than in non-dyslexic children, independently of the attentional task; in the condition without vibration, the attentional performance of dyslexics was significantly impaired with respect to the non-dyslexic group of children [38]. According to Nicolson and Fawcett [39], this evidence suggests that the cerebellum, which is responsible for the integration of proprioceptive inputs during balance, could be impaired in the dyslexic population.

In the present study, we compared postural capabilities in a group of dyslexic and non-dyslexic children while they were asked to perform two types of saccadic eye movements (visually guided saccades and voluntary saccades while reading a text silently). Recall that attention is strictly linked to the execution of saccadic eye mouvements [40] and that several structures of the central nervous system in the cerebral cortex (frontal, parietal, occipital) and in the brainstem (paramediane pontine reticular formation and the superior colliculus) play an important role in the postural control [41] as well as in the saccadic eye movement control [42]. Based on these findings, one could expect an interference between oculomotor and postural control. Note that several studies have showed the effect of saccadic eye movement on postural control in adults but none explored this issue in children. For instance, an improvement of postural stability with saccadic eye movements was found [43]–[47]. In contrast others [48]–[50] found that saccades increased postural instability. The interest for studing oculomotor tasks together with posture in dyslexic population comes from the fact that studies dealing with oculomotor performance in dyslexics showed poor oculomotor control only during oculomotor cognitive task (as reading) but not during simple oculomotor task as is the case for visually guided saccades [51]–[54].

Our initial hypothesis was that, in accordance with the U-shaped non-linear interaction model [4], the two oculomotor tasks could influence postural control in dyslexic and non-dyslexic 10-year-old children in different ways given that oculomotor performance and postural capabilities are different in the two groups of children.

## Materials and Methods

### Subjects

Eighteen dyslexic children participated in the study. The dyslexic children were recruited from the pediatric hospital where they were referred for a complete evaluation of their dyslexia with an extensive examination including neurological/psychological and phonological capabilities. For each child, the time required to read a text, text comprehension, and the ability to read words and pseudowords were evaluated with the L2MA battery [55]. This is the standard test developed by the applied psychology centre in Paris, and is used everywhere in France. Inclusion criteria were scores on the L2MA more than 2 standard deviations from the mean and a normal mean intelligence quotient (IQ, evaluated with the WISC-IV), namely between 80 and 115. The mean age of the dyslexic children was 10.3±0.8 years, the mean IQ was 100±7 and the mean reading age was 8±1 years. The dyslexic children had no signs of hyperactivity or developmental coordination disorder (DCD). Diagnostic and Statistical Manual of Mental Disorders Fourth Edition (DSM-IV) was used to exclude hyperactive children [56]. Dyslexic children were also screened for DCD with the movement assessment battery for children (M-ABC) and their score was above the 21th percentile. A carefully selected age-matched control group (mean age: 10.5±1 years) of 18 non-dyslexic children was chosen. These children had to satisfy the following criteria: no known neurological or psychiatric abnormalities, no history of reading difficulty, and no visual stress or difficulties with near vision. IQ and reading measurements were not available for these children, but their scores for French (reading, comprehension, spelling), mathematics and foreign languages were all beyond the mean scores for the class. Recruitment of controls based on school performance alone has been used by other researchers [57]–[59].

Both non-dyslexic and dyslexic children underwent an ophthalmological examination accompanied by orthoptic evaluation of their visual functions (median values shown in Table 1).

**Table 1:** Clinical characteristics of dyslexic and non-dyslexic children.

	TNO	NPC	Phoria Far	Phoria Near	Div. Far	Div. Near	Conv. Far	Conv. Near
**Dyslexic children**	40	3	ortho	ortho	4	10	17	30
**Non-dyslexic children**	40	3	ortho	Exo 2	4	16*	20	30

Clinical characteristics of all children tested. Median values for binocular vision (stereoacuity test, TNO measured in seconds of arc); near point of convergence (NPC measured in cm); heterophoria at far and near distance, measured in prism diopters; ortho = orthophoria; exo = exophoria; vergence fusional amplitudes (divergence and convergence) at far and near distance, measured in prism diopters. Asterisks indicate that the value is significantly different for the group of dyslexic children (p<0.01).

Visual acuity was normal (≥20/20) for all children in both groups. All children had normal binocular vision (60 seconds of arc or better), as evaluated with the TNO random dot test. The near point of convergence was normal for both groups of children tested (≤5 cm). Moreover, an orthoptic evaluation of vergence fusion capability using prisms and Maddox rod was carried out at far and near distances. The phoria (i.e., latent deviation of one eye when the other eye is covered, using the cover-uncover test) was normal for all children tested. At far distance, the divergence amplitudes were similar in both groups of children examined. In contrast, at near distance, the divergence amplitudes were significantly smaller in the dyslexic group than in the non-dyslexic children. An ANOVA showed a significant main effect of group (F_(1,34)_ = 6.50, p<0.01). Convergence amplitudes at both far and near distance were similar for dyslexic and non-dyslexic children.

In sum, the orthoptic evaluation showed a tendency toward poor divergence fusional capabilities in dyslexic children.

The investigation adhered to the principles of the Declaration of Helsinki and was approved by our Institutional Human Experimentation Committee (CPP Ile de France I, Hôpital Hotel-Dieu). Written consent was obtained from the children's parents after an explanation of the experimental procedure.

### Platform

A platform (principle of strain gauge) consisting of two dynamometric clogs (standards by Association Française de Posturologie, produced by TechnoConcept, Céreste, France) was used to measure postural stability. The position of the feet was as follows: heels 4 cm apart and the feet spread out symmetrically at an angle of 30° with respect to the child's sagittal axis. Arms were vertically along the body. The excursions of the center of pressure (CoP) were measured for 25.6 seconds and the surface of the CoP was calculated following the standards proposed by Gagey et al. [60]; the equipment included a 16-bit analog-digital converter. The sampling frequency of the CoP was 40 Hz.

### Stimuli

Visual stimuli were presented on a flat screen (1280×768 pixels), placed 40 cm from the children. The elevation of the screen was adjusted as a function of the height of each child so that its center exactly faced the eyes. Two visual tasks were used for stimulation: a visually guided saccade task and silent reading of a text.

Visually guided horizontal and vertical saccades were elicited by using a simultaneous paradigm to induce reflexive saccades. At the start of each trial, a central black square of 1.4° was switched on for a period of 1500 ms; afterwards the square was switched off, and simultaneously a target (little green man, smiley) of 1.4° appeared at the periphery of the screen (eccentricity of the target was 10° to right or left, up or down) and stayed on for 1500 ms. Children were invited to make a horizontal or vertical saccade to the target. A total of 9 saccades were stimulated for the postural recording. It should be noted that eye movements have not been recorded, consequently we were unable to quantify oculomotor responses.

For the reading task, a six-line test was presented to the child. The mean character width was 0.5° and the text was written in black Courier font on a white background. The paragraph was extracted from *Monsieur Petit*, a text produced by ELFE (Cogni-Sciences, www.cognisciences.com), which allows for a rapid evaluation of the reading capabilities of children aged 7 to 12. We did not give any instructions for reading but we simply asked to the child to read silently. Silent reading was chosen for two reasons: firstly to avoid inducing anxiety in the dyslexic group, secondly to avoid any neck, abdomen and chest muscles activity that are well known to affect postural control [61]–[62].

Reading task induced mainly horizontal saccades even if some oblique saccades are necessary to start the new line, while with the visually guided paradigm used in our task child has to make both horizontal as well as vertical saccades. Note that Rougier & Garin [47] did not find different effect on posture between these two types of saccade direction. However, it will be interesting to explore further this issue.

Finally, in order to evaluate the reading score, after the recording we asked each child what was the last word he/she had read. The total number of words read in the two different paragraphs was counted.

### Postural recording procedure

The child stood on the platform, in front of the screen located 40 cm away from him/her. Postural measurements were made while the child engaged in horizontal and vertical saccades and read a text silently. The child was instructed to stay as stable as possible, with the arms along the body and to look at the screen in order to perform the saccades or read the text. Each condition was performed twice; the order of the two conditions varied randomly between the children. Each postural recording test was followed by a rest lasting for a few minutes.

### Data analysis

The posture measurement method is similar to that used in a previous study [63]. We analyzed the surface area and mean speed of the CoP excursion. Surface area is a good measure of CoP spatial variability [64] and mean speed represents a good index of the amount of neuromuscular activity required to regulate postural control [65]–[66].

Statistical analysis comprised two-way ANOVAs with group of children (dyslexics and non-dyslexics) as between-subject factor and condition (saccades and reading) as within-subject factor. The effect of a factor is significant when the p-value is below 0.05.

## Results

**Figure 1:**
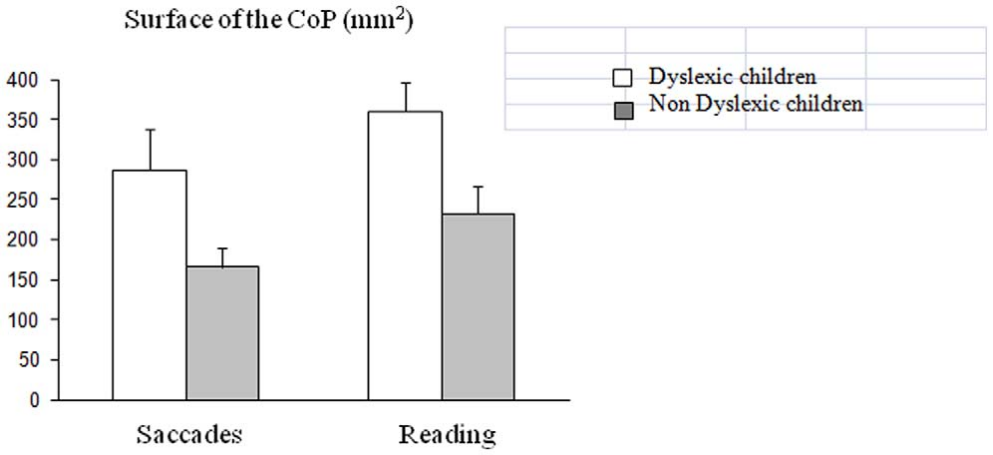
Means and standard deviations for surface area of CoP in the saccade and reading tasks for the two groups of children (dyslexic and non-dyslexic).

Figures show the postural parameters (surface area and mean speed of the CoP) that were measured during the two experimental conditions for dyslexic and non-dyslexic children. Concerning the surface of the CoP (Figure 1), the ANOVA showed a significant effect of group (F_(1,34)_ = 8.57, p<0.006); non-dyslexic children were more stable than dyslexic children. The ANOVA also; showed a significant effect of condition on the surface of the CoP, which was systematically smaller when children were making saccades than when they were reading a text (F_(1,34)_ = 5.64, p<0.02). There are no significant interactions between group of children and condition.

**Figure 2:**
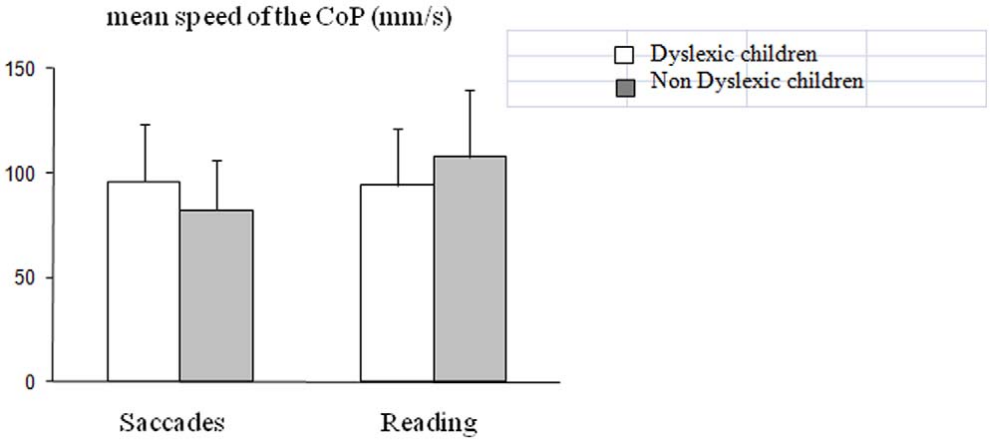
Means and standard deviations for mean speed of CoP in the saccade and reading tasks for the two groups of children (dyslexic and non-dyslexic).

Figure 2 shows the data obtained concerning the mean speed of the CoP. The ANOVA showed a significant interaction between group of children and condition (F_(1,34)_ = 4.85, p<0.03): post hoc comparisons showed that the mean speed of the CoP during reading was significantly greater than during saccades only for the non-dyslexic children (p<0.01).

The reading scores for both groups of children were also evaluated by counting the number of words read in the two postural measures. The ANOVA showed a significant difference between the two groups of children (F_(1,34)_ = 9.31, p<0.004); the non-dyslexic children read a larger number of words than the dyslexic children (80±8 and 49±18, respectively).

## Discussion

The goal of the study was to compare the effect of a dual task on postural stability in a group of dyslexic and non-dyslexic children. The main findings are as follows: (i) independently of the task, dyslexic children are more unstable than non-dyslexic children; (ii) reading impairs postural stability more than a saccade task in both groups of children. These findings will be discussed individually below.

### Poor postural stability in dyslexic children

Several studies have examined postural stability in dyslexic children but the finding that these subjects have balance impairment is still controversial. Indeed, as mentioned in the Introduction, some authors have hypothesized that balance deficits in the dyslexic population are related to other developmental deficits such as attention-deficit/hyperactivity disorder and developmental coordination disorder [35], [67]. Note, however, that a recent study [68] reported poor postural control in children and adults with dyslexia even without comorbid attention deficits. Pioneering studies [32], [69] suggest that the balance deficits observed in the dyslexic population could be due to an impairment affecting the processing of sensory information by the cerebellum. This hypothesis is based on the finding that dyslexics were impaired in both attentional and balance capabilities when two tasks were performed simultaneously [36]. Subsequent studies have also reported poor postural stability during a dual task in dyslexic children, providing more evidence for the cerebellar hypothesis [28]–[29]. Studies examining posture in children with cerebellar deficits [70]–[71] have reported poor postural stability, suggesting a difficulty of these children to integrate multimodal sensory information for balance control and/or a difficulty in properly compensate the deficit of sensory input [41]. Based on our findings, we could assume that dyslexic children are not able to use sensorial inputs in order to assure good postural control particularly when they have to perform a dual task. This hypothesis is in line with the study of Barela et al. [72].

The present data, from dyslexic children without hyperactivity/developmental coordination deficits, are in line with the work of Quercia et al. [64]; indeed, dyslexic children show a significantly larger surface of the CoP during a dual task than non-dyslexic children of comparable age, while the mean speed of the CoP was similar in both groups of children (at least while making saccades). Recall that the mean speed of the CoP, according to Geurts et al. [65] and Maki et al. [66], is believed to reflect the muscular energy used by the body for self-stabilization. Thus, dyslexic children do not seem to use a speed strategy to override their instability more than non-dyslexics do. This finding is new, and needs to be explored further in studies dealing with postural control in different types of dual task in dyslexic and non-dyslexic children in order to better understand whether and how dyslexics are able to use their energy to achieve better balance control.

### Reading versus saccading: different effects on postural stability

The second important result of the present study is the different effects of saccadic and reading tasks on postural stability in dyslexic and non-dyslexic children. Indeed, both groups of children showed a greater surface area of the CoP during reading than during the saccade task. Reading is a higher cognitive activity that depends on multiple processes (perception, eye movements, and linguistic/semantic capacities); consequently, during reading, cognition and postural control may require the same mechanisms. Given the difficulty of this task, children might shift their attention towards the reading task, instead of postural stability, leading to poor balance. This occurs for dyslexic and non-dyslexic children, at least for the surface area of the CoP. Interestingly, the results also showed that only non-dyslexic children significantly increased the mean speed of the CoP during reading, suggesting that unlike the dyslexic group, they are able to use their muscular effort to control their equilibrium when they are given a highly cognitively demanding dual task. Dyslexic children did not show this capability, indeed mean speed of CoP did not change in two conditions. Such finding could suggest in dyslexic population a deficit in allocating the muscular energy required for stabilizing the body.

Taken together, all these data are in line with the U-shaped non-linear interaction model [4], which posits that the type of cognitive task can influence postural stability. Thus, we suggest that, in children (both dyslexic and non-dyslexic at about 11 years of age), the more demanding the cognitive task, the worse the postural sway. This has also been reported in previous work [17], [20] on non-dyslexic children of similar ages, for whom different cognitive tasks (counting backward or Stroop-type task) led to impaired postural stability. In contrast, these results did not confirm previous findings [16] in which children between 8 and 10 years old changed their postural control strategies by increasing the attentional demand. Note, however, that the results of Blanchard et al.'s [16] study, showing strong interference between cognition and posture, revealed a discordant effect on postural measures depending on the parameter taken into account (sway variability or length of CoP path).

### Conclusion

In conclusion, this study, in accordance with previous reports, provided evidence suggesting that dyslexic children have impaired postural stability compared to non-dyslexic children of similar age. Furthermore, a reading task impaired postural control more than a saccadic task; most likely, the attention needed for the reading task leads to poor postural control in both groups of children. The increase in the surface of the CoP in the absence of any increase in the mean speed of the CoP reported in the dyslexic group is in agreement with the hypothesis of sensorimotor theory where the sensory or motor deficit could lead to a multimodal integration difficulties in the cerebellum. These findings could be explored further with eye movement recordings.
